# Acute kidney injury after hip fracture surgery among elderly patients in the ICU: incidence, risk factors and their predictive value, clinical impact—a retrospective single-center study

**DOI:** 10.1080/0886022X.2025.2560595

**Published:** 2025-09-24

**Authors:** Jun Wang, Lifang Wang, Ying Bai, Hao Wang

**Affiliations:** ^a^ Department of Critical Care Medicine, Beijing Jishuitan Hospital, Capital Medical University, Beijing, China; ^b^ Department of Clinical Epidemiology, Beijing Jishuitan Hospital, Capital Medical University, Beijing, China

**Keywords:** Hip fracture, elderly patient, acute kidney injury, Intensive care unit, surgery, risk factor

## Abstract

**Objective:**

To explore the incidence, risk factors and clinical effects of acute kidney injury (AKI) in elderly patients with hip fracture admitted to the ICU postoperatively and assess the predictive value of risk factors.

**Methods:**

We retrospectively analyzed consecutive elderly patients with hip fracture who underwent surgery and were admitted to the ICU at Beijing Jishuitan Hospital, Capital Medical University (October 2022–August 2023). Patients were divided into AKI group and non-AKI group according to whether AKI occurred. Demographic, preoperative, intraoperative, and postoperative data were compared. Multivariate logistic regression identified AKI risk factors, and receiver operating characteristic (ROC) curves evaluated their predictive ability.

**Results:**

Among 156 patients, 31 (19.9%) developed AKI. Multivariate logistic regression analysis showed that female sex, intraoperative blood transfusion, postoperative albumin level and postoperative Acute Physiology and Chronic Health Evaluation (APACHE) II score were independent risk factors for AKI (*p* < 0.05). ROC curve showed that the combination of these four indicators and postoperative APACHE II score could effectively predict AKI occurrence, and the area under the curve (AUC) was 0.93 and 0.86, respectively (*p* < 0.05). Patients in the AKI group stayed in ICU longer than those in the non-AKI group (*p* < 0.05).

**Conclusion:**

The incidence of AKI was high in elderly patients with hip fracture admitted to ICU after surgery. The combination of being female, receipt of intraoperative blood transfusion, postoperative albumin level and postoperative APACHE II score had good predictive value for AKI. The occurrence of AKI resulted in prolonged ICU stay.

## Introduction

With the progress of population aging, hip fracture in the elderly has become a worldwide problem, leading to significant morbidity and mortality. The global number of hip fractures is expected to increase to 4.5 million by the year 2050 [1].

Currently, surgery is the main treatment method for elderly hip fracture. However, due to the characteristics of elderly patients with advanced age and multiple morbidty, as well as the stimulation of intraoperative anesthesia and surgical trauma, the incidence of postoperative complications is high, which brings a large medical and economic burden to the family and society [2,3]. AKI is one of the common complications, which may lead to poor prognosis [4–11].

In recent years, many studies have focused on AKI after hip fracture in elderly patients. The current reports show the incidence of AKI in the elderly after hip fracture surgery is 9.8–20.6% [4–11]. Most of these studies were conducted in the orthopedic ward, and we believe that the incidence of AKI in patients admitted to the ICU after surgery should be different from that in the orthopedic ward.

In this study, elderly patients with hip fracture admitted to ICU after surgery were taken as the research objects to evaluate the occurrence, risk factors and clinical effects of AKI, and explore methods to predict the occurrence of AKI. It provides basis for clinicians to screen high-risk groups of AKI and take corresponding preventive measures as early as possible.

The study was approved by the Ethics Committee of Beijing Jishuitan Hospital, Capital Medical University (No. K2024 − 390 − 00). Because of the retrospective nature of the study, the Ethics Committee waived the consent requirement.

## Material and methods

### Study design and population

We retrospectively analyzed consecutive elderly patients with hip fracture who underwent surgery and were admitted to the ICU of Beijing Jishuitan Hospital, Capital Medical University, from October 2022 to August 2023. Inclusion criteria were as follows: 1) patients above 65 years old (including 65 years old); 2) hip fractures including femoral neck fractures, intertrochanteric fractures and subtrochanteric fractures; 3) surgical treatment after fracture, including internal fixation and artificial hip replacement; 4) patients who returned to ICU directly from operating room after operation. The exclusion criteria were as follows: 1) patients with AKI before admission to ICU; 2) patients with previous chronic kidney disease (CKD) or after kidney transplantation or nephrectomy; 3) patients with fracture caused by tumor.

### Data collection

The following data were collected for each study object: 1) demographic information including biologic sex, age, body mass index (BMI), fracture type and preoperative comorbidities; 2) preoperative laboratory index including hemoglobin, albumin, blood glucose and N-terminal pro-brain natriuretic peptide (NT-pro BNP); 3) intraoperative information including anesthesia method, type of surgery, duration of operation, intraoperative blood transfusion and intraoperative hypotension; 4) postoperative data including albumin, NT-pro BNP, postoperative and preoperative NT-pro BNP difference, acute physiology and chronic health evaluation (APACHE) II score and sequential organ failure assessment (SOFA) score; 5) prognostic information including length of stay (LOS), LOS in ICU and hospitalization cost; 6) indicators used to diagnose AKI including baseline serum creatinine, creatinine and urine volume on day 1–3 after admission to ICU.

### Relevant definitions and diagnostic criteria

AKI was diagnosed according to the Kidney Disease Improving Global Outcomes (KDIGO) criteria [12]. The severity classification of AKI (AKI stage 1, 2, and 3) was also based on this criteria. Postoperative AKI was defined as the occurrence of AKI that met the KDIGO criteria within the first 72 h after ICU admission in this study. The baseline serum creatinine (Scr) is defined as the lowest value among: 1) the patient’s Scr values measured within 7 days before ICU admission, compared with the Scr level at ICU admission; or 2) the average of Scr values from the year prior to hospitalization and the 7 days before admission. For patients admitted emergently with abnormal Scr at admission (no prior CKD history) or when baseline Scr is unavailable, the baseline value is estimated using the Modification of Diet in Renal Disease (MDRD) equation assuming that baseline estimated glomerular filtration rate (eGFR) is 75 mL/min per 1.73 m^2^, as recommended by the Acute Disease Quality Initiative (ADQI) [13].

### Statistical analysis

SPSS 26.0 software was used for statistical processing. We performed univariate analysis using Students t test for continuous numerical variables satisfying a normal distribution, the chi-square or Fisher exact test for categorical variables, and the Kruskal–Wallis test or Mann–Whitney test for continuous variables that did not meet a normal distribution. Variables with statistical differences in univariate analysis were placed into multivariate analysis. Multivariate logistic regression analysis was performed to identify independent risk factors for developing AKI in our study population. The predictive efficacy of risk factors for AKI was analyzed by ROC curve. A *p*-value of less than 0.05 (two-sided) was considered statistically significant.

## Results

### AKI incidence

Based on the inclusion and exclusion criteria, a total of 156 patients were finally enrolled in the study. Of these, 31 patients were diagnosed with AKI. The incidence of AKI was 19.9%. Among them, there were 19 cases (61.3%) in AKI stage 1, 11 cases (35.5%) in AKI stage 2, and 1 case (3.2%) in AKI stage 3. 1 patient (3.2%) has undergone renal replacement therapy.

**Figure 1.**
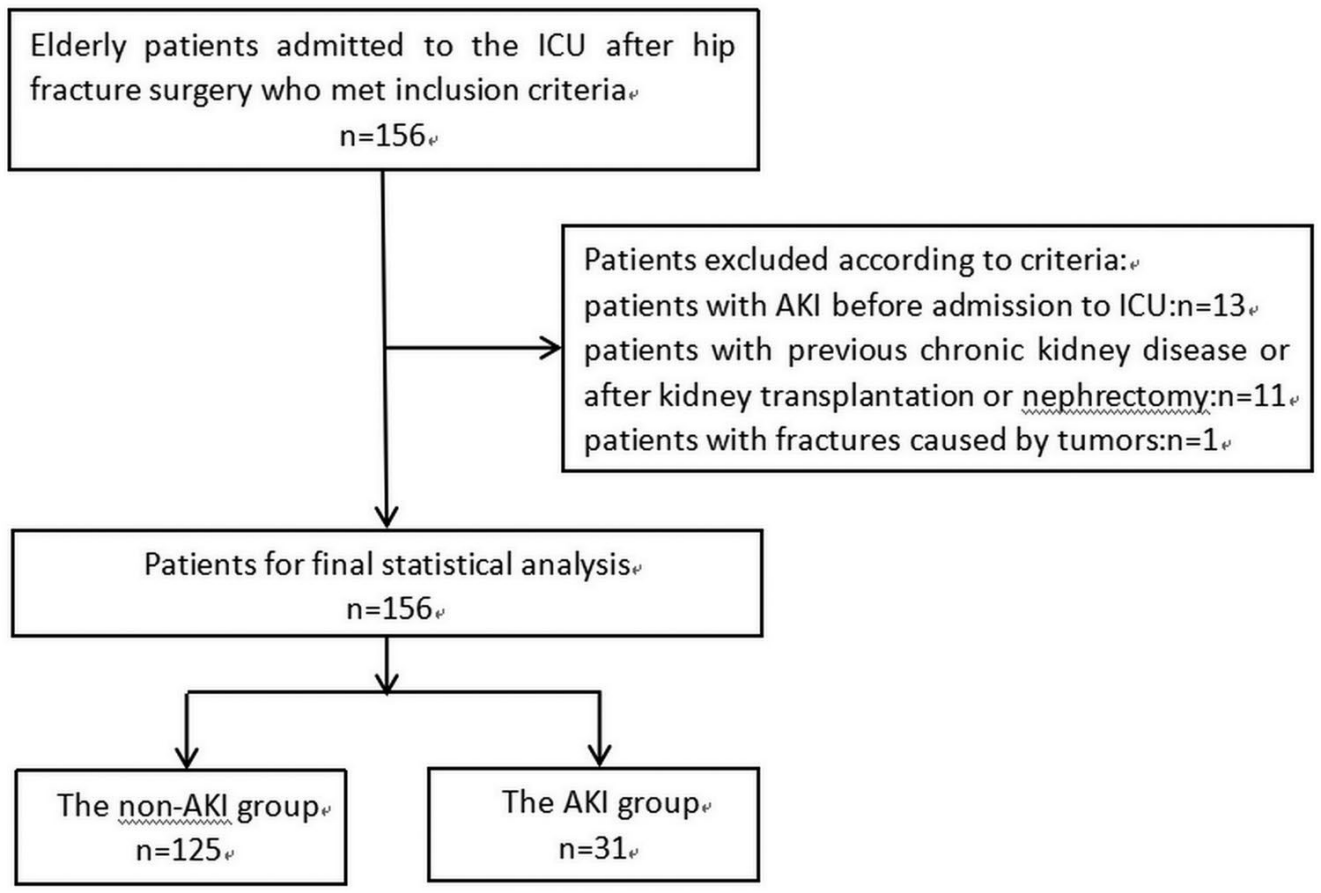
Flow chart.

Thus, two groups of patients were formed: the non-AKI group—125 patients and the AKI group—31 patients (Figure 1).

### Patient demographics

Demographic data of our patients is presented in Table 1. There were significantly more female patients in the AKI group: 27 (87.1%) versus 65 (52.0%) in the non-AKI group (*p <* 0.01). Proportion of diabetic patients was significantly higher in the AKI group: 13 (41.9%) versus 30 (24.0%) in the non-AKI group (*p* = 0.04). Similarly, proportion of chronic heart failure patients was significantly higher in the AKI group: 5 (16.1%) versus 5 (4.0%) in the non-AKI group (*p* = 0.04). It is worth noting that age and BMI were not significantly different between the two groups in our study.

**Table 1.** Demographic data.

Variable	Non-AKI group *N* = 125	AKI group *N* = 31	*p*-value
Age, years	86 (81, 90)	88 (79, 92)	0.29
Sex			< 0.01
Male	60 (48.0%)	4 (12.9%)	
Female	65 (52.0%)	27 (87.1%)	
BMI, Kg/m^2^	22.6 ± 3.9	23.3 ± 4.9	0.12
Fracture type			0.63
Femoral neck fractures	59 (47.2%)	12 (38.7%)	
Intertrochanteric fractures	64 (51.2%)	18 (58.1)	
Subtrochanteric fractures	2 (1.6%)	1 (3.2)	
Comorbidities	109 (87.2%)	27 (87.1%)	1.00
Hypertension	71 (56.8%)	22 (71.0%)	0.15
Diabetes mellitus	30 (24.0%)	13 (41.9%)	0.04
Coronary heart disease	39 (31.2%)	12 (38.7%)	0.43
Chronic heart failure	5 (4.0%)	5 (16.1%)	0.04
Arrhythmia	21 (16.8%)	4 (12.9%)	0.59
Chronic lung disease	11 (8.8%)	4 (12.9%)	0.72
Chronic liver disease	4 (3.2%)	0 (0.0%)	0.56
Dementia	7 (5.6%)	1 (3.2%)	0.94
Previous cerebrovascular disease	32 (25.6%)	4 (12.9%)	0.13
Other chronic diseases	10 (8.0%)	5 (16.1%)	0.30

BMI: body mass index.

### Preoperative characteristics

Preoperative data of patients from two groups is presented in Table 2. There were no significant differences except for preoperative hemoglobin. Level of preoperative hemoglobin was significantly lower in the AKI group: 9.9 (9.3, 11.2) versus 11.0 (9.5, 12.4) in the non-AKI group (*p* = 0.01).

**Table 2.** Preoperative data.

Variable	Non-AKI group *N* = 125	AKI group *N* = 31	*p*-value
Preoperative hemoglobin, g/dL	11.0 (9.5, 12.4)	9.9 (9.3, 11.2)	0.01
Preoperative albumin, g/dL	3.7 ± 0.4	3.8 ± 0.4	0.61
Preoperative blood glucose, mmol/L	7.3 (6.2, 9.4)	7.4 (6.2, 9.0)	0.87
Preoperative NT-pro BNP, pg/ mL	590.0 (294.4, 1269.0)	759.0 (365.0, 1920.0)	0.10

NT-pro BNP: N-terminal pro-brain natriuretic peptide.

### Intraoperative characteristics

Information about the intraoperative course of the patients is presented in Table 3. Significantly more patients had blood transfusions during surgery in the AKI group: 16 (51.6%) versus 32 (25.6%) in the non-AKI group (*p* < 0.01). Type of anesthesia, type of surgery, operation duration and proportion of patients with intraoperative hypotension were not significantly different between the two groups.

**Table 3.** Intraoperative data.

Variable	Non-AKI group *N* = 125	AKI group *N* = 31	*p*-value
Anesthesia method			0.69
General anesthesia	9 (7.2%)	1 (3.2%)	
Non-general anesthesia	116 (92.8%)	30 (96.8%)	
Type of surgery			0.59
Hip arthroplasty	51 (40.8%)	11 (35.5%)	
Internal fixation	74 (59.2%)	20 (64.5%)	
Duration of operation, min	74.0 (60.0, 100.0)	60.0 (60.0, 90.0)	0.06
Intraoperative blood transfusion	32 (26.6%)	16 (51.6%)	< 0.01
Intraoperative hypotension	1 (0.8%)	1 (3.2%)	0.36

### Postoperative characteristics

Patients’ postoperative data is presented in Table 4. Level of postoperative albumin was significantly lower in the AKI group: 2.9 (2.7, 3.1) versus 3.2 (2.9, 3.4) in the non-AKI group (*p* < 0.01). Postoperative NT-pro BNP was significantly higher in the AKI group: 1127 (670, 3566) versus 760 (335, 1780) in the non-AKI group (*p* = 0.01). Patients in the AKI group had significantly higer APACHE II score on admission to ICU: 11 (10, 12) versus 8 (7, 9.5) in the non-AKI group (*p* < 0.01). They also had significantly higer SOFA score on admission to ICU: 3 (2, 4) versus 2 (1, 3) in the non-AKI group (*p* = 0.02).

**Table 4.** Postoperative data.

Variable	Non-AKI group *N* = 125	AKI group *N* = 31	*p*-value
Postoperative albumin, g/dL	3.2 (2.9, 3.4)	2.9 (2.7, 3.1)	< 0.01
Postoperative NT-pro BNP, pg/ mL	760.9 (335.3, 1780.0)	1127.0 (670.0, 3566.0)	0.01
NT-pro BNP difference, pg/ mL	49.0 (−94.5, 346.8.0)	135.7 (−166.0, 1152.0)	0.49
APACHE II score	8 (7, 9.5)	11 (10, 12)	< 0.01
SOFA score	2 (1, 3)	3 (2, 4)	0.02

NT-pro BNP: N-terminal pro-brain natriuretic peptide; APACHE: Acute Physiology and Chronic Health Evaluation; SOFA: Sequential Organ Failure Assessment.

### Potential risk factors for AKI

Variables with statistical differences in the above univariate analysis, including sex, diabetes, chronic heart failure, preoperative hemoglobin, intraoperative blood transfusion, postoperative albumin, postoperative NT-pro BNP, APACHE II score and SOFA score at ICU admission, were placed into multivariate logistic regression analysis. The results are presented in Table 5. Female sex was found to be a significant independent risk factor for AKI development, OR 5.12 (95% CI:1.15–22.86). There was a significantly independent correlation between intraoperative blood transfusion and AKI development, OR 5.13 (95% CI:1.05–25.03). Level of postoperative albumin was independently correlated with AKI significantly, OR 0.55 (95% CI:0.40–0.77). APACHE II score was also found to be a significant independent risk factor for AKI development, OR 2.02 (95% CI:1.39–2.93).

**Table 5.** Results of multivariate logistic regression analysis.

Variable	Odds ratio	95% CI	*p*-value
Sex	5.12	1.15–22.86	0.03
Diabetes mellitus	1.76	0.45–6.85	0.42
Chronic heart failure	1.20	0.10–14.54	0.89
Preoperative hemoglobin	1.01	0.96–1.07	0.65
Intraoperative blood transfusion	5.13	1.05–25.03	0.04
Postoperative albumin	0.55	0.40–0.77	< 0.01
Postoperative NT-pro BNP	1.00	1.00–1.00	0.1
APACHE II score	2.02	1.39–2.93	< 0.01
SOFA score	0.99	0.67–1.47	0.96

NT-pro BNP: N-terminal pro-brain natriuretic peptide; APACHE: Acute Physiology and Chronic Health Evaluation; SOFA: Sequential Organ Failure Assessment.

### Predictive value of risk factors for AKI

**Figure 2.**
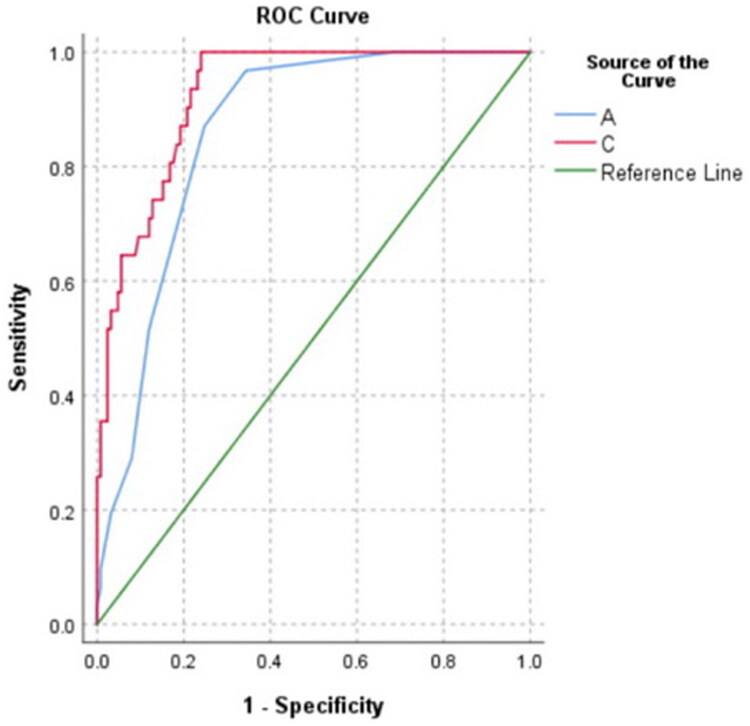
ROC curves of APACHE II score and combined index predicting AKI.

ROC curve was used to evaluate the predictive value of the above risk factors for AKI (Table 6). Among the individual indicators, APACHE II score had the highest prediction efficiency for AKI, AUC 0.86 (95% CI: 0.80–0.92). The combination of these four indicators also had high predictive value for AKI, AUC 0.93 (95% CI: 0.89–0.97). As the ROC analysis shows (Figure 2), there was a statistical difference between APACHE II score and the combined index in predicting AKI, AUC difference 0.07 (*p* < 0.01).

**Table 6.** Predictive value of risk factors for AKI.

Variable	AUC	95% CI	*p*-value
Sex	0.68	0.58–0.77	< 0.01
Intraoperative blood transfusion	0.63	0.52–0.74	0.03
Postoperative albumin	0.21	0.13–0.29	< 0.01
APACHE II score	0.86	0.8–0.92	< 0.01
Combined index	0.93	0.89–0.97	< 0.01

APACHE: acute physiology and chronic health evaluation.

A: APACHE II score; C: Combined index.

### LOS, LOS in ICU and hospitalization cost

We used univariate analysis of variance to compare the clinical effects of AKI in elderly patients after hip fracture surgery (Table 7). Patients in the AKI group remained in the ICU significantly longer, 3 (2, 4) days versus 2 (1, 3) days in the non-AKI group (*p* = 0.01).

**Table 7.** Data of clinical effects.

Variable	Non-AKI group *N* = 125	AKI group *N* = 31	*p*-value
LOS, day	7 (6, 9)	7 (6, 10)	0.83
LOS in ICU, day	2 (1, 3)	3 (2, 4)	0.01
hospitalization cost, yuan	65187 (58584, 75781)	69469 (60843, 90306)	0.14

LOS: length of stay; ICU: intensive care unit.

## Discussion

To the best of our knowledge, few studies have been conducted on AKI in the elderly after hip fracture surgery in the ICU. Our study showed that the incidence of AKI in elderly patients admitted to ICU after hip fracture surgery was as high as 19.9%. Female sex, intraoperative blood transfusion, low level of postoperative albumin and high APACHE II score after surgery were independent risk factors for AKI. Both the combination of these four indicators and APACHE II score after surgery can effectively predict the occurrence of AKI. Postoperative AKI prolonged ICU stay.

In recent years, many relevant studies have shown that the incidence of postoperative AKI in the elderly with hip fracture ranges from 9.8 to 20.6% [4–11]. It can be seen that the incidence of AKI in our study is within this range and on the high side. There are two possible reasons for the high incidence of AKI in our study population. Firstly, patients who are admitted to ICU after surgery are sicker and more likely to develop postoperative complications, including AKI, than those in the general ward. Secondly, in our study, both creatinine and urine volume were used as diagnostic criteria to improve the diagnosis rate of AKI. Many previous studies used only creatinine as a criterion, which may have led to missed diagnosis.

Blood transfusion is common in major surgery, which may affect the postoperative morbidity and mortality of patients. A retrospective study by Piyush et al. showed that in elderly patients undergoing hip fracture surgery, the incidence of postoperative AKI was 2.8 times higher in patients with intraoperative transfusion than in those without transfusion [14]. While in our multivariate analysis, it was indicated that intraoperative blood transfusion was an independent risk factor for postoperative AKI. Patients requiring intraoperative blood transfusion often meet blood loss and hypovolemia, which may lead to renal hypoperfusion. In addition, immune modulation associated with blood transfusion may also activate inflammatory responses systematically, including in the kidney. Both hypoperfusion and inflammation are pathophysiological mechanisms that cause AKI.

As shown in our findings, postoperative low albumin level was significantly associated with AKI. Kyun-Ho et al. explored the relationship between early hypoalbuminemia and postoperative AKI following hip fracture surgery. They reported that the minimal early postoperative serum albumin level < 2.9 g/dL at any point during the first two postoperative days was an independent risk factor for AKI [15]. The protective effect of albumin on the kidney is manifested in maintaining colloid osmotic pressure, increasing adequate circulating volume, promoting increased renal blood fow, and preserving renal function [16]. We believe that serum albumin is more important for the elderly with poor renal reserve.

APACHE II score is commonly used to assess the severity of the condition in critically ill patients. Patients with a high APACHE II score often have more and serious comorbidities, unstable vital signs after surgery, internal environment disorders and so on. And they are at greater risk for postoperative complications including AKI. A prospective observational study found that APACHE II score was an independent risk factor for postoperative AKI in critically ill patients following cardiac surgery [17]. Our study population, elderly critically ill patients after hip fracture surgery, had similar results.

Female sex was a risk factor for AKI after hip fracture surgery in our patients, but age was not associated with postoperative AKI. Previous studies have also explored the relationship between these demographic factors and postoperative AKI, and the results have been inconsistent [4–11,14–15]. We tend to attribute this inconsistency to patients’ heterogeneity and geographical differences. Patients with comorbidities of diabetes and chronic heart failure have poor renal reserve, and are more likely to develop AKI after surgery. There was a higher proportion of patients with diabetes and chronic heart failure in our AKI group, but there was no statistical difference in multivariate analysis. We believe that the correlation between these two indicators and AKI can be further explored by increasing the sample size.

Furtherly, ROC curves showed that postoperative APACHE II score had a high predictive value for postoperative AKI in elderly patients with hip fractures, with an AUC of 0.86. We believe that this reflects the impact of previous physical health, age, and the overall state of the patient after surgery on the kidney. According to our results, the combination of being female, receipt of ntraoperative blood transfusion, postoperative albumin level and postoperative APACHE II score were more predictive of postoperative AKI than postoperative APACHE II score alone. We can easily obtain these four indicators in clinical diagnosis and treatment, and then use their combination to predict the occurrence of postoperative AKI.

In our hospital’s specialized geriatric hip fracture unit, surgery is scheduled as soon as possible after admission. The length of surgery is minimized, so there is little general anesthesia and little mechanical ventilation. Patients are discharged as soon as possible after surgery. Some of them have gone home, some to rehabilitation facilities, and fewer patients have died in our hospital. Our study showed that the AKI group had longer ICU stay time. Although the length of hospital stay and hospitalization costs were not statistically different between the two groups, the AKI group had a relatively long hospital stay and a relatively high hospitalization cost.

## Study limitations

Our study was a single-center, retrospective study. At the trial design stage, we thought that preoperative hemoglobin, postoperative NT-pro BNP and postoperative SOFA score would be independent risk factors for postoperative AKI. However, the results of multivariate analysis didn’t support this assumption. We are inclined to conduct future multicenter, prospective studies to further explore the relationship between these indicators and AKI. In addition, long-term prognostic indicators such as mortality and incidence of CKD were not counted, which is also the focus of our future research. Moreover, the simple method of determining baseline creatinine may affect the diagnosis rate of AKI. Some studies on AKI have used a variety of methods to determine baseline creatinine [18]. The MDRD formula we used is a commonly used method. We also used the diagnostic criteria of urine volume, which is helpful to improve the diagnostic accuracy. Finally, we did not include the patients who developed AKI beyond day 3. According to our observations in clinical work, 3 days after hip fracture surgery in the elderly is a period with a relatively high incidence of AKI, and AKI that occurs after 3 days is rare. However, in order to enhance rigor, in the prospective studies to be conducted in the future, we will attempt to include AKI after 3 days.

## Conclusion

Our study focused on AKI in elderly patients admitted to ICU after hip fracture surgery. We found that the incidence of AKI may be relatively high in these patients. The combination of female sex, intraoperative blood transfusion, postoperative albumin level and postoperative APACHE II score may be a simple and effective new method to predict AKI. This method can be used to guide clinicians to screen high-risk groups for AKI and implement renal protection strategies early to reduce the occurrence of AKI and related deaths. Further prospective, large sample studies are needed to verify the feasibility of this approach in clinical practice.
